# Distinct patterns of neurodegeneration after TBI and in Alzheimer's disease

**DOI:** 10.1002/alz.12934

**Published:** 2023-01-25

**Authors:** Neil S.N. Graham, James H. Cole, Niall J. Bourke, Jonathan M. Schott, David J. Sharp

**Affiliations:** ^1^ Department of Brain Sciences Imperial College London London UK; ^2^ UK Dementia Research Institute Centre for Care Research and Technology at Imperial College London London UK; ^3^ Dementia Research Centre UCL Queen Square Institute of Neurology London UK; ^4^ Centre for Medical Image Computing UCL London UK; ^5^ Centre for Injury Studies Imperial College London London UK

**Keywords:** Alzheimer's, atrophy, dementia, head injury, neurodegeneration, TBI

## Abstract

**Introduction:**

Traumatic brain injury (TBI) is a dementia risk factor, with Alzheimer's disease (AD) more common following injury. Patterns of neurodegeneration produced by TBI can be compared to AD and aging using volumetric MRI.

**Methods:**

A total of 55 patients after moderate to severe TBI (median age 40), 45 with AD (median age 69), and 61 healthy volunteers underwent magnetic resonance imaging over 2 years. Atrophy patterns were compared.

**Results:**

AD patients had markedly lower baseline volumes. TBI was associated with increased white matter (WM) atrophy, particularly involving corticospinal tracts and callosum, whereas AD rates were increased across white and gray matter (GM). Subcortical WM loss was shared in AD/TBI, but deep WM atrophy was TBI‐specific and cortical atrophy AD‐specific. Post‐TBI atrophy patterns were distinct from aging, which resembled AD.

**Discussion:**

Post‐traumatic neurodegeneration 1.9–4.0 years (median) following moderate‐severe TBI is distinct from aging/AD, predominantly involving central WM. This likely reflects distributions of axonal injury, a neurodegeneration trigger.

**Highlights:**

- We compared patterns of brain atrophy longitudinally after moderate to severe TBI in late‐onset AD and healthy aging.
- Patients after TBI had abnormal brain atrophy involving the corpus callosum and other WM tracts, including corticospinal tracts, in a pattern that was specific and distinct from AD and aging.
- This pattern is reminiscent of axonal injury following TBI, and atrophy rates were predicted by the extent of axonal injury on diffusion tensor imaging, supporting a relationship between early axonal damage and chronic neurodegeneration.

## 1 INTRODUCTION

Traumatic brain injury (TBI) is a risk factor for dementia and progressive neurodegeneration.^1^ It may be particularly amenable to early disease‐modifying treatment to reduce later‐life dementia risk.^2^ A substantial proportion of all dementia cases have been attributed to TBI,^1^ including increased risk of Alzheimer's disease (AD) specifically.^3^, ^4^, ^5^ Dementia risk remains elevated for many decades after the injury,^6^ with evidence of a dose‐response relationship.^7^ A range of neuropathologies has been reported to occur after TBI, with axonal injury considered to play a central role in the generation of proteinopathies of hyperphosphorylated tau and amyloid β.^8^ These can be seen in the chronic phase following injury and have been associated with features of neurodegeneration, such as progressive brain atrophy.^9^, ^10^, ^11^ However, it is unclear whether patterns of atrophy are similar after TBI, in comparison with AD and healthy aging.

A powerful way to compare the chronic effects of TBI relative to other neurodegenerative diseases and brain aging is to use magnetic resonance imaging (MRI) to measure spatial pattern atrophy. Longitudinal volumetric T1‐weighted MRI assessment of brain volume can quantify atrophy locations and rates, indicating the extent and distribution of neuronal loss, the end product of diverse degenerative pathways.^12^ Gross atrophy, particularly of WM, has long been recognized following TBI,^13^ and longitudinal neuroimaging shows elevated atrophy rates in the chronic phase in vivo, indicating progressive degeneration years after injury.^14^ Post‐traumatic WM atrophy is particularly prevalent in large fiber bundles such as the corpus callosum, internal and external capsules, and inferior and superior longitudinal fasciculi.^14^, ^15^, ^16^ These tracts are highly susceptible to traumatic axonal injury,^17^, ^18^ which predicts the extent and pattern of WM degeneration.^19^

The progression of brain atrophy in typical late‐onset AD often follows a stereotypical spatiotemporal course, initially involving the hippocampi before spreading to involve temporal lobes more widely, parietal lobes, and frontal regions later in the disease. Primary motor and somatosensory cortices are typically preserved.^20^ This atrophy reflects neuronal loss^21^, ^22^ and is related predominantly to the distribution and extent of tau deposition, rather than of amyloid pathology.^23^, ^24^

Healthy aging is also associated with progressive loss of brain tissue. MRI studies suggest a frontotemporal cortical preponderance of age‐related volume loss, though a key question is the extent to which this is an artifact of unidentified presymptomatic AD cases.^25^ To address this, healthy older adults at very low risk of AD, defined using genetic and biomarker approaches, were assessed.^26^ In these groups, compared with mild cognitive impairment and AD patients, there was considerable overlap, yet prefrontal cortical changes appeared more specific to aging.

Here we compare progressive brain atrophy between TBI (2 to 4 years following moderate to severe injury), AD, and healthy aging. To our knowledge, this study represents the first effort at doing so and will help to clarify the nature of atrophy early into the chronic phase, which may reflect variable contributions of slow Wallerian or toxic spreading proteinopathy. This is important given the epidemiological link between TBI and AD.^3^ We test whether the progressive brain atrophy triggered by TBI is distinct from that observed in AD and healthy aging. We hypothesized that (1) longitudinal patterns of atrophy in the chronic phase after moderate to severe TBI has a WM preponderance distinct from AD and that (2) this is distinct from healthy aging. We used serial T1‐weighted MRI to quantify brain volume changes in gray matter (GM), WM, and cerebrospinal fluid (CSF) regions, as well as voxelwise, and performed group comparisons to define spatial similarities and differences between TBI, AD, and healthy aging.

**RESEARCH IN CONTEXT**

1. **Systematic Review**: The authors used PubMed to identify previous longitudinal volumetric MRI studies assessing chronic neurodegeneration after TBI, in AD and aging. In vivo patterns of progressive atrophy have not previously been compared between the two conditions.
2. **Interpretation**: We found evidence of a distinct pattern of abnormal, progressive central white matter (WM) atrophy after moderate to severe TBI, which is specific, and not present in AD, which had distinctive cortical involvement. The magnitude of brain atrophy rates was similar to that in healthy participants three decades older than the TBI group, though atrophy patterns at baseline were distinct between TBI and aging and were reminiscent of axonal injury distributions.
3. **Future Directions**: Large‐scale longitudinal studies, including acute biomarker characterization at the time of injury, with prolonged follow‐up, molecular‐specific, and volumetric imaging, in addition to serial cognitive testing, would help clarify the relationship between TBI, brain aging, and AD.

## 2 METHODS

### 2.1 Study participants

Participants were recruited across two research sites as part of two separate research programs. AD patients were recruited as part of an MRI test‐retest reliability cohort at University College London (UCL), with data from this study previously published and available.^12^, ^27^, ^28^, ^29^, ^30^ TBI patient data were collected as part of an ongoing program of neuroimaging research at Imperial College London (ICL). Data were combined to provide the maximum possible interscan intervals and reduce noise. The TBI program previously published on post‐traumatic atrophy^14^, ^19^ as well as trauma‐associated diffusion tensor imaging abnormalities.^17^, ^31^, ^32^ No comparisons have been made with other neurodegenerative diseases or aging.

Participant groups and baseline characteristics are shown in Table 1, including participants with TBI, and two groups of healthy controls, one matched in age to the TBI population, the other age‐matched to the AD population. Ethical approvals were granted by the relevant research ethics committees across the sites. Patients with AD were members of the Minimal Interval Resonance Imaging in Alzheimer's Disease (MIRIAD) dataset acquired by UCL.^30^ This included 46 subjects with mild to moderate probable AD defined using National Institute of Neurological and Communicative Diseases and Stroke/Alzheimer's Disease and Related Disorders Association (NINCDS‐ADRDA) criteria,^33^ with volumetric T1‐weighted MRI collected longitudinally (range 0.46–2.07 years) and all imaging acquired on the same 1.5T Signa scanner (GE Medical Systems, Milwaukee, WI) alongside age‐matched controls. There was no history of significant TBI in any AD participant. A single MIRIAD participant was excluded due to a very short interscan interval under 2 months (>5 months was used as a cut‐off to reduce noise). Patients with AD were a median of 68.6 years old (IQR 9.9) and well matched to healthy volunteers with a median age of 68.5 years (IQR 7.5). Mini‐Mental Status Examination (MMSE) was performed at baseline in the AD group, with a median score of 19 (IQR 9.5), within the moderate range.

**TABLE 1:** Overview of study participants

	**Alzheimer's disease**	**Healthy controls**	**Traumatic brain injury**	**Healthy controls** (younger)	**Healthy controls** (older)
Participants, *N*	45	23	48	23	15
Age, years, median (IQR)	68.6 (9.9)	68.5 (7.5)	40.0 (17.2)	35.0 (19.7)	65.8 (7.3)
Male, *N* (%)	18 (40%)	12 (52%)	39 (81%)	13 (57%)	14 (93%)
Interscan interval, years, median (IQR)	1.49 (0.98)	1.49 (0.99)	2.1 (2.1)	1.1 (0.4)	1.7 (0.5)
Scanner site	UCL		ICL		

Abbreviations: UCL, University College London; ICL, Imperial College London; IQR, interquartile range.

Forty‐eight patients after moderate to severe TBI were assessed at ICL comprising a longitudinal cohort of patients in the chronic phase (>5 months) after moderate to severe TBI, defined by the Mayo classification.^34^ Criteria for inclusion in the moderate to severe category comprise any specific abnormalities in neuroimaging (e.g., contusion, subdural and extradural hematomas, and brainstem injury), penetrating injuries, worst Glasgow Coma Scale < 13 in the first 24 h, post‐traumatic amnesia lasting more than 24 h or loss of consciousness greater than 30 min. Patients after TBI were a median of 40 years old at baseline (IQR 17.2), which was 1.9 years (median) after injury (IQR 6.3). Injuries were typically sustained in road traffic accidents (44%) or falls (29%), followed by assaults (15%). Most TBI patients (92%) had a period of post‐traumatic amnesia. MRI scans showed focal lesions in 69% of cases, and 46% (21/46) had microhemorrhages on MRI, suggestive of diffuse vascular injury. Patients had a range of post‐injury cognitive impairments as previously described, including of memory, processing speed, and executive function.^14^

MIRIAD was originally established to assess MRI reliability, so all of these subjects were repeatedly reimaged, typically nine times over approximately 2 years. A proportion (*n* = 25) of the TBI patients had three scans, due to participation in the ICL TBI neuroimaging program. Nineteen of the healthy controls at ICL were imaged on three occasions, owing to their participation in a test‐retest imaging reliability study. For all participants with more than two scans available, the scan pair with the longest interscan interval was chosen to reduce noise in the generation of atrophy rates.

To control for the effect of age and scanner, three groups of healthy volunteers were assessed longitudinally. Twenty‐three healthy controls were assessed at UCL with a median age of 68.5 (IQR 7.5). At ICL, 23 healthy controls (HCs) age‐matched to the TBI patients were assessed (median age 35 years, IQR 19.7). To define atrophy patterns associated with aging, 15 older HCs with no history of head injury or cognitive problems were also assessed at ICL (median age 65.8 years, IQR 7.3).

There was no significant difference in age between AD patients and HCs at UCL or between TBI patients and the younger group of HCs at ICL. As expected, the older group of HCs at ICL were not significantly different in age from AD patients or the UCL healthy control group, but they were older than the TBI age‐matched HCs (*W* = 0, *r* = 0.84, *p* < 0.001) at ICL. The proportion of females differed significantly across the five groups (X^2^ = 24.4, df = 4, *V* = 0.40, *p* < 0.001). There were no sex differences between the AD patients and age‐matched controls; however, the TBI group had a lower proportion of females than the age‐matched HC group (X^2^ 4.9, d = 1, *V* = 0.26, *p* = 0.028) but not the aging group.

### 2.2 Image acquisition

Data were acquired using slightly different MR acquisition parameters on two different scanners, across the two research sites (1.5T GE system at UCL using IR‐FSPGR, 3T Siemens at ICL using an MPRAGE sequence): Technical details are supplied in the Supplementary Methods. Each patient was, however, imaged longitudinally on the same system, allowing within‐subject analyses that were not confounded by scanner differences. These differences have been shown not to significantly influence volumetric analyses in multicenter studies.^35^

### 2.3 Neuroimaging processing

**FIGURE 1:**
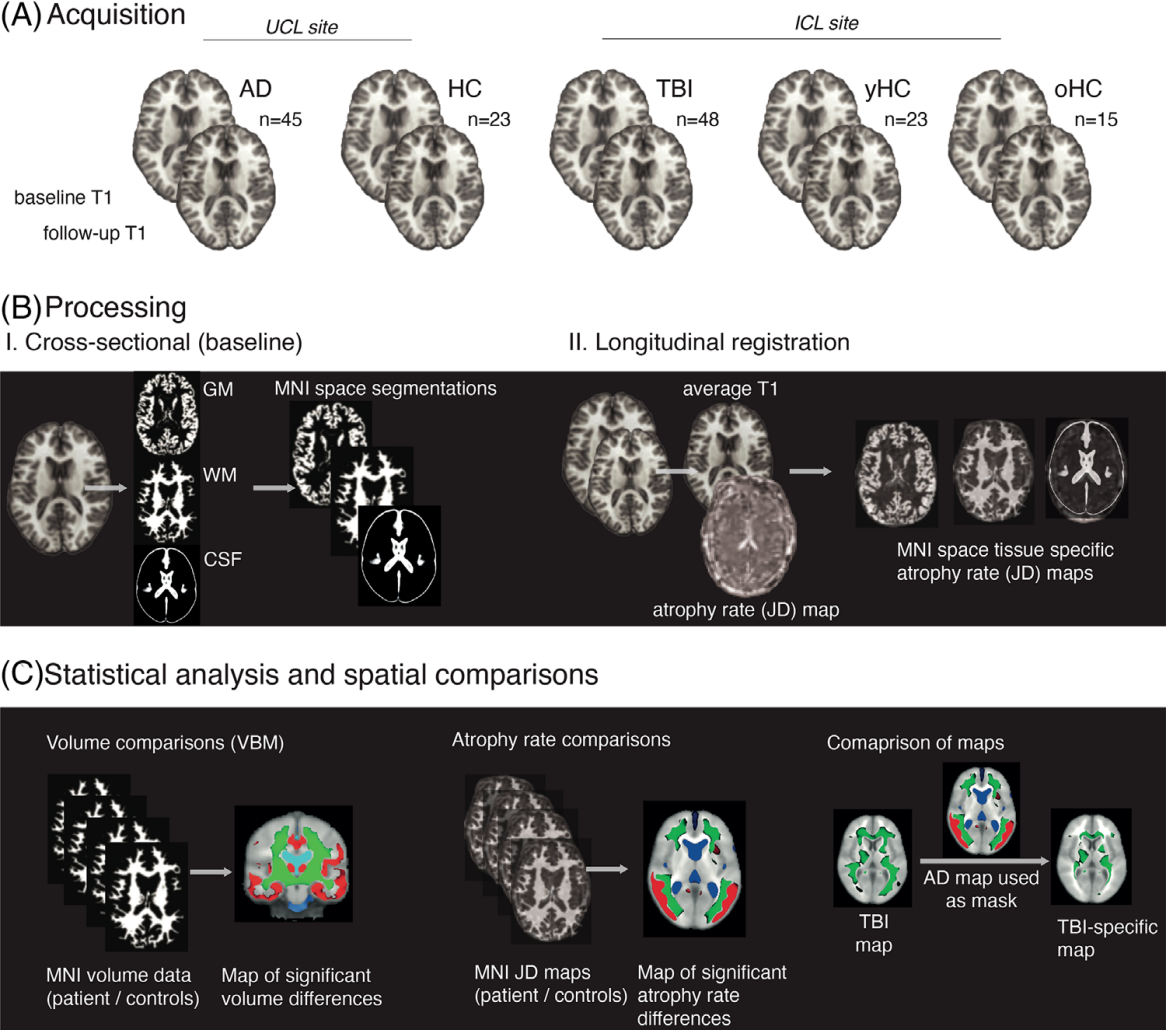
Neuroimaging methods. (A) Serial volumetric T1‐weighted MRIs were acquired in (Alzheimer's disease) AD and age‐matched healthy controls (HCs) at University College London; patients after a single moderate to severe traumatic brain injury (TBI), young TBI age‐matched HCs (yHC), and older HCs (oHC, matched to the AD group) at ICL. (B) Images were processed using SPM12 with segmentation and nonlinear registration to MNI standard space. Images were modulated to preserve volume information. Longitudinal registration of scan pairs generated temporal average space T1 maps and Jacobian determinant (JD) rate maps of atrophy rates per subject. The JD map was multiplied by the segmented temporal average image and registered (nonlinearly) to MNI space. (C) MNI space images were used in permutation testing (FSL) to assess for voxelwise differences in baseline volume or atrophy rate, in different tissue classes. Subtractive analyses, using *p*‐statistic images outputted from other disease entities, show disease‐specific or shared patterns of progressive atrophy.

An established pipeline was used to analyze volumetric T1‐weighted MRI data in SPM12 (UCL)^36^ (Figure 1). Briefly, T1‐weighted images at baseline and follow‐up were segmented into GM, WM, and CSF, with volumes calculated. Quality was confirmed visually. Longitudinal pairwise registration was performed, whereby each individual's baseline T1 was iteratively coregistered to the follow‐up image, producing a midpoint “subject average‐space” image. When more than two scans were available for any study participant within the two cohorts, the scan pair with the longest interscan interval was chosen.

For each subject a three‐dimensional voxelwise “Jacobian determinant” map was produced showing the rate of expansion/contraction to move from baseline to follow‐up. The subject‐average T1 was then segmented into GM, WM, and CSF, thresholded and binarized to produce individualized average‐space masks, allowing sampling of mean Jacobian determinant (JD) values for each tissue class. These JD values are multiplied by 100 to produce an annualized percentage of brain volume change rates. The JD maps were next registered to standard space: Each patient's JD map was multiplied by the relevant subject‐average space tissue image (GM, WM, and CSF), producing an individualized tissue‐specific JD map. A study average‐space template was generated using a random selection of 40 average‐space T1 images, weighted evenly across patients and controls across the two sites, and used within the SPM nonlinear DARTEL tool.^37^ Flow fields capturing voxelwise deformations were generated from subject‐average space images to the study template, followed by final affine registration to Montreal Neurological Institute (MNI) space. These flow fields were applied to the baseline images and tissue‐specific JD maps, resulting in standard space tissue‐specific atrophy rate and volume maps.

Neuroimaging contrasts were conducted using the general linear model and permutation testing (Randomize, FSL 6.0, 10,000 permutations) with age, sex, and total intracranial volume as nuisance covariates, other than for assessing aging in controls at ICL, where age was not regressed out.^38^ Voxelwise multiple comparisons correction was performed with threshold‐free cluster enhancement (TFCE).

To produce disease‐specific longitudinal atrophy maps, TFCE‐corrected *p* value maps for each condition were binarized using a cut‐off of 0.9. Regions of significant change were those with a one‐sided *t* test with *p* < 0.05, reflecting the strong a priori hypothesis that each condition would be associated with varying degrees of GM atrophy, WM atrophy, and CSF expansion, relative to controls. An AD‐specific longitudinal atrophy map was generated by multiplying the AD TFCE *p*‐map by an inverted mask of the TBI map; the opposite approach was taken to generate a TBI‐specific map. The AD‐specific map was subtracted from the AD map to produce a map of shared atrophy in TBI and AD. The degree of spatial overlap between two mask images was quantified by the Dice coefficient (twice the common area between the scans, divided by the total number of voxels in each mask image) using MATLAB (Mathworks, R2017b).

Diffusion tensor imaging (DTI) analysis methods are described in the Supplementary Methods.

### 2.4 Statistical analyses

Analyses on summary measures were performed using R studio (R version 3.6.0). Normal variables were compared with Student's *t* test and non‐normal variables with nonparametric methods, for example, Wilcoxon rank sum test. False discovery rate (FDR) correction was used for post hoc testing. Chi‐squared tests were used for categorical variables. Owing to the strong prior of greater atrophy in aging versus healthy controls, TBI versus healthy controls, and AD versus controls, one‐sided *t* tests were used for these voxelwise comparisons with a significance level of *p* < 0.05. All other tests were two‐sided. Z‐scoring of FA and JD in TBI patients was performed by subtracting each raw value from the control healthy control mean and dividing this by the healthy control standard deviation. Effect sizes were calculated using the rstatix package (version 0.7.0).

## 3 RESULTS

### 3.1 Lower baseline volumes of GM and WM in AD and after TBI

**FIGURE 2:**
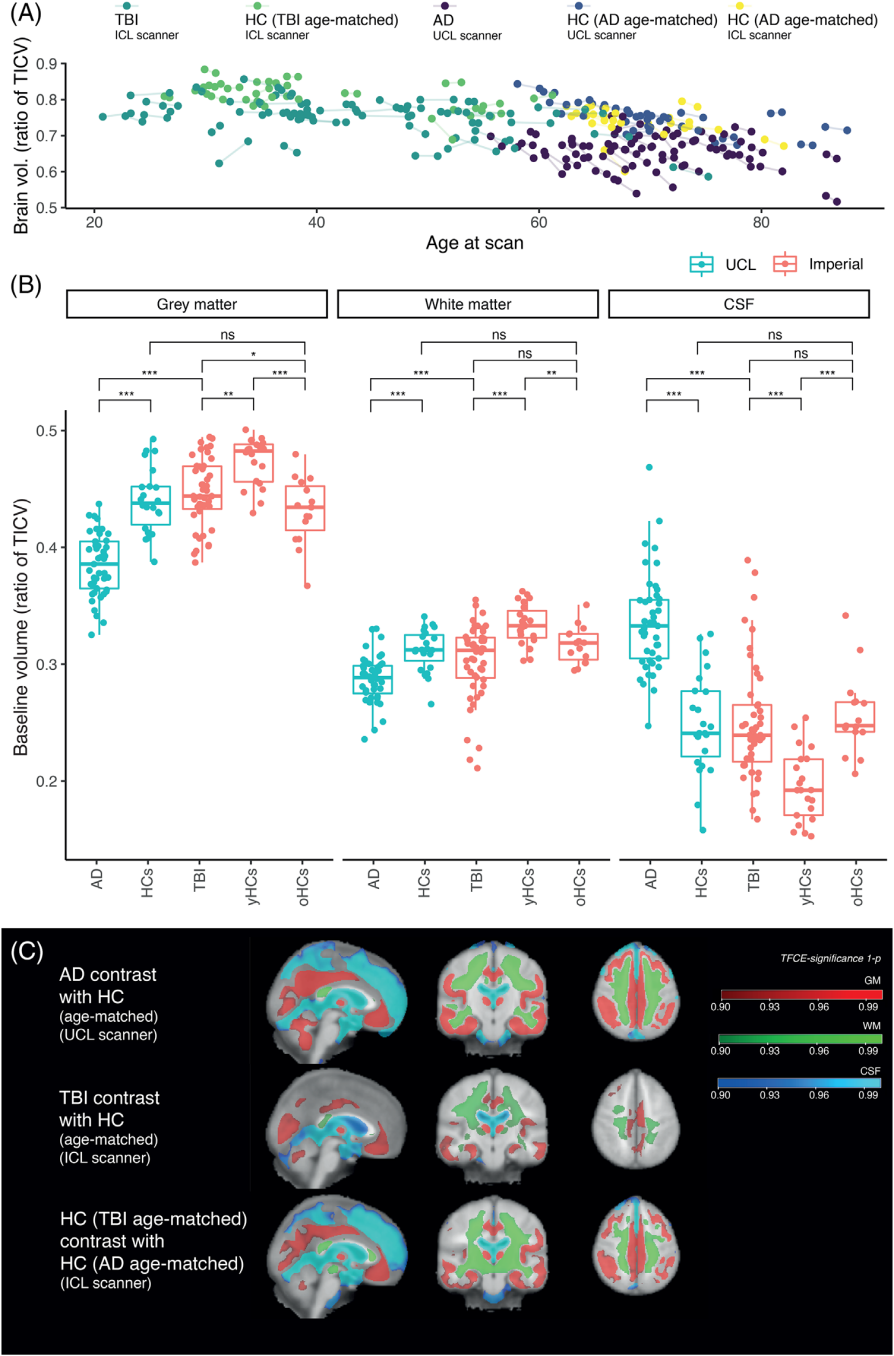
Brain volumes and baseline atrophy patterns. (A) Normalized (divided by total intracranial volume [TICV]) whole‐brain volumes (defined as white plus gray matter volume) related to age at MRI scanning and participant group. (B) Brain volumes across different groups normalized by TICV. Comparisons are between AD and HCs at UCL; AD and TBI; TBI and ICL young healthy controls (yHCs); TBI and ICL older healthy controls (oHCs); yHCs and oHCs; HCs at UCL and oHCs at ICL. Wilcox tests performed; *p* values are false discovery rate (FDR) corrected for multiple comparisons. *p < 0.05; **p < 0.01; ***p < 0.001. (C) Voxelwise contrasts (threshold‐free cluster enhancement corrected *p* value significance maps) demonstrate patterns significant for gray matter (red) and white matter (green) atrophy and cerebrospinal fluid expansion (blue).

Patients with AD had the lowest volumes of GM and WM and the largest CSF volume (Figure 2B, FDR‐corrected, all *p* values < 0.001; Table 2). TBI patients had significantly lower baseline volumes than HCs in WM (TBI median WM to total intracranial volume [TICV] ratio 0.31, HC 0.33, *r* = 0.50, *p* < 0.001) and GM (TBI 0.45; HC median 0.48, *r* = 0.37, *p* = 0.006), with larger CSF volume (0.24 vs. 0.19, *r* = 0.55, *p* < 0.001). There was no significant difference of WM, GM, or CSF after TBI versus with older controls.

**TABLE 2:** Brain volumes at baseline and atrophy rates over time in patients and healthy controls

	**Alzheimer's disease**	**Healthy volunteers (UCL)**	**Traumatic brain injury**	**Healthy volunteers (ICL, TBI age‐matched)**	**Healthy volunteers (ICL, AD age‐matched)**
Baseline volume, median (IQR)					
Gray matter	0.38 (0.04)	0.44 (0.04)	0.45 (0.04)	0.48 (0.02)	0.43 (0.04)
White matter	0.29 (0.03)	0.31 (0.02)	0.31 (0.03)	0.33 (0.02)	0.32 (0.02)
Whole brain	0.67 (0.05)	0.76 (0.06)	0.76 (0.05)	0.81 (0.05)	0.75 (0.03)
CSF	0.33 (0.05)	0.24 (0.06)	0.24 (0.05)	0.19 (0.05)	0.25 (0.03)
JD percentage brain volume change rate, median (IQR)					
Gray matter	−0.8 (0.8)	−0.2 (0.2)	−0.2 (0.5)	0.0 (0.5)	0.0 (0.4)
White matter	−0.9 (0.9)	−0.2 (0.3)	−0.3 (0.7)	0.1 (0.7)	−0.4 (0.6)
Whole brain	−0.9 (0.9)	−0.2 (0.3)	−0.3 (0.5)	0.1 (0.6)	−0.2 (0.4)
CSF	1.0 (0.7)	0.3 (0.3)	0.4 (0.9)	0.3 (0.6)	0.9 (0.4)

Abbreviations: ICV, intracranial volume; JD, Jacobian determinant atrophy rate.

Voxelwise patterns of baseline brain volume and CSF expansion were assessed. AD and TBI patients were compared with their own age‐matched controls, acquired on the same scanner (Figure 2B). Older and younger healthy controls (ICL groups) were compared to assess change related to healthy aging.

In AD, significantly lower volume was evident in a wide number of regions, particularly including temporal cortices, hippocampus, amygdala and parahippocampal gyri, cingulate gyri, and parietal association cortices. There was atrophy but to a lesser a lesser extent frontally, involving superior, middle, and inferior frontal gyri. There was no substantial change in pre/postcentral gyri. There were widespread reductions in WM volume as well as expansion of the lateral ventricles. Volume reductions were spatially similar in healthy aging to AD, though the aging contrast showed less extensive regional temporal lobe loss.

Lower baseline brain volume in the TBI group was predominantly in WM regions. There was extensive WM atrophy when TBI patients were compared to age‐matched controls: This was seen most prominently within the precentral gyri, descending corticospinal tracts, superior longitudinal fasciculi, and corpus callosum. Patients after TBI had limited regional GM atrophy involving inferior temporal gyri, hippocampi, parahippocampal gyri, orbitofrontal, and cingulate gyri in particular. There were no significant differences between TBI and aging at baseline.

### 3.2 Longitudinal atrophy rates are raised in AD and after TBI

**FIGURE 3:**
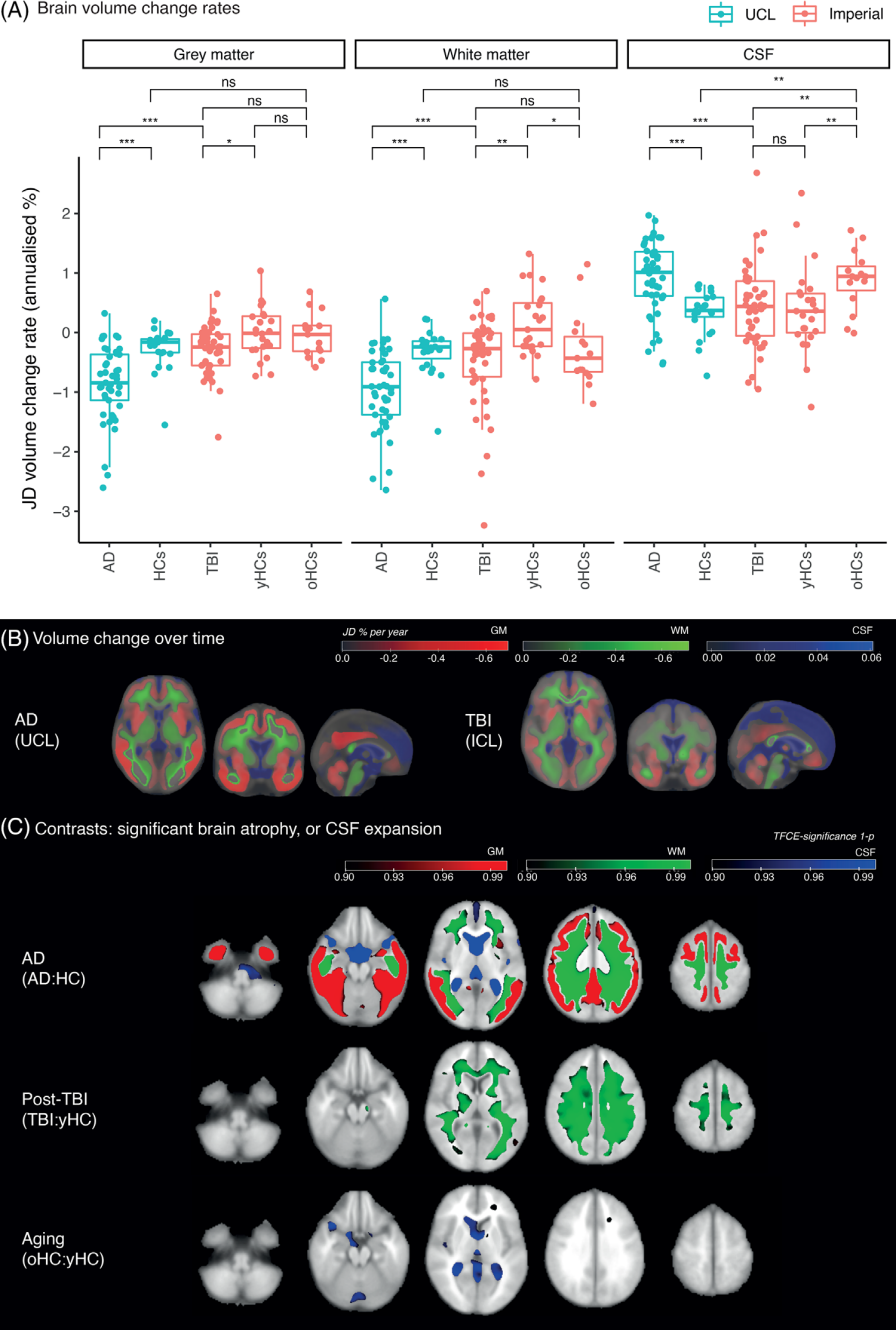
Longitudinal patterns of brain volume change in Alzheimer's disease and after traumatic brain injury. (A) JD (Jacobian determinant) volume change rates in different tissue classes. Negative values: contraction; positive: expansion; HC: healthy control. (B) Group average JD maps showing atrophy rates in gray matter (red) or white matter (green) and cerebrospinal fluid (CSF) expansion (blue). (C) Voxelwise contrasts of JD rate maps. Regions with significantly greater longitudinal atrophy rates are shown in red for gray matter and green for white matter, and those with significant expansion in CSF are in blue.

Patients with AD had markedly raised atrophy rates in GM (median JD percentage volume change rate in AD 0.8 vs. HC 0.2, *r* = 0.50) and WM (–0.9 vs. –0.2, *r* = 0.50) and higher rates of CSF expansion (1.0 vs. 0.3; *r* = 0.48, all *p* values < 0.001) in comparison with age‐ and scanner‐matched HCs (Table 2, Figure 3A).

Atrophy rates were abnormally elevated in the WM of TBI patients and were significantly higher than age‐ and scanner‐matched controls (median WM JD –0.3 vs. 0.1, *r* = 0.35, *p* = 0.019) but were similar to older HCs (median WM JD –0.4). Similarly, GM atrophy rates were significantly raised after TBI versus age‐matched controls (TBI –0.2 vs. HC 0.0, *r* = 0.31, *p* = 0.05), but were not different from older HCs. CSF expansion rates did not differ between TBI and age‐matched controls but were significantly higher in older HCs than TBI patients (TBI median 0.4 vs. older HC 0.9, *r* = 0.34, *p* = 0.040).

Directly comparing AD and TBI showed that, in AD, atrophy rates were higher in both GM (AD –0.8 vs. –0.2, *r* = 0.50, *p* < 0.001) and WM (–0.9 vs. –0.3, *r* = 0.42, *p* < 0.001), with a raised rate of CSF expansion in AD (1.0 vs. 0.4, r = 0.37 *p* = 0.003). Assessing changes in atrophy rates associated with aging, that is, comparing older with younger HCs (scanner‐matched), older controls had greater CSF expansion rates (0.9 vs. 0.3, *r* = 0.43, *p* = 0.040) but no significant differences in either WM or GM atrophy rates.

### 3.3 Longitudinal atrophy patterns in AD and TBI

Group average atrophy rate maps are shown for AD and TBI (Figure 3B). A comparison of patients with AD and age‐matched HCs revealed increased GM atrophy, particularly of the fusiform, parahippocampal, inferior, middle, and superior temporal gyri, cingulate, angular gyri, middle frontal gyri, and frontal poles. AD patients did show WM atrophy, including of superior and inferior longitudinal fasciculi. Areas not showing increased atrophy included primary motor cortices and parts of the WM, including the corticospinal tracts (Figure 3C, upper row).

Comparison of TBI with age‐matched controls revealed increased atrophy rates in a large number of WM regions, including the corpus callosum, pyramidal tracts, and superior and inferior longitudinal fasciculi (Figure 3C, middle row). There was no significant difference in GM atrophy. Comparing older and younger controls revealed a small region of increased atrophy in older individuals in the left frontal WM, including the anterior thalamic radiation, with increased rates of CSF expansion present (Figure 3C, lower row).

### 3.4 Distinct patterns of progressive atrophy are seen in AD and after TBI

**FIGURE 4:**
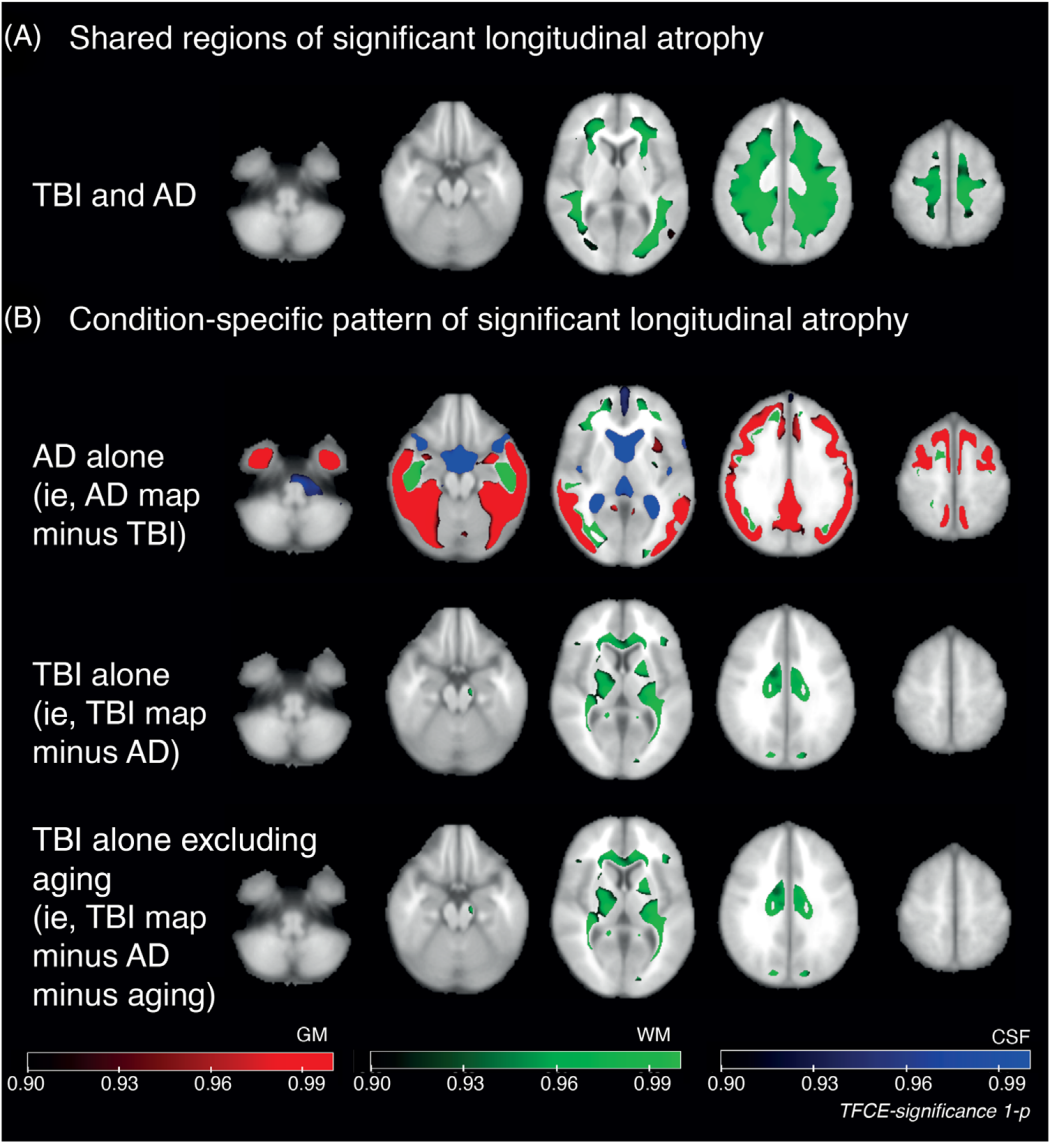
Comparisons of atrophy rates across Alzheimer's disease (AD) and traumatic brain injury (TBI). (A) Composite map showing shared regional significant volume change over time in TBI and AD. (B) Composite maps of shared significant volume change over time: top map—regions of significant change found only in AD but not in TBI; middle map—regions significant in TBI only; lower map—as per middle, but with regions significantly changing in aging also excluded.

To define progressive atrophy patterns specific to each of AD and TBI, maps of significant regional brain volume change over time were compared (Figure 4). There was substantial overlap between the AD and TBI maps across parts of the WM, indicated by a high Dice coefficient of 0.80, but no overlap in the GM (Dice = 0.00) or CSF (Dice = 0.00). Spatially, overlapping regions were predominantly subcortical rather than involving structures of the deep WM (Figure 4A).

As expected, given the few regions surviving multiple‐comparison correction on the voxelwise map of significant longitudinal volume change, there was little overlap when comparing TBI and aging (GM Dice = 0.00, WM Dice = 0.03, CSF DICE = 0.00). Aging and AD had overlap in CSF (Dice = 0.61), but less in WM (Dice = 0.31) or GM (Dice = 0.00).

An “AD‐specific” atrophy map was generated by subtracting regions of significant TBI‐related atrophy from the AD map. This showed raised atrophy rates specific to AD within the temporal GM, parietal and occipital cortices, and WM regions, including the inferior occipitofrontal fasciculi bilaterally, which are not seen after TBI (Figure 4B, upper row).

Using the same approach, a “TBI‐specific” atrophy map (Figure 4B, middle row) was generated by subtracting the AD image from the TBI map. This showed TBI‐specific increases in atrophy of the corona radiata, corpus callosum, internal capsules, corticospinal tracts, and cerebral peduncle (right). Finally, for completeness, this TBI‐specific map with respect to AD was refined by removing regions of significant change attributable to aging. The resulting image appeared substantially unchanged (Figure 4B, lower row).

### 3.5 Atrophy rates after TBI are predicted by extent of diffuse axonal injury but not time since injury

We previously showed that WM tracts are affected by diffuse axonal injury, indicated by reduced fractional anisotropy (FA), show greater longitudinal atrophy.^19^ Baseline DTI measures were available for *n* = 46 (96%) of TBI patients and *n* = 22 (96%) age‐matched HCs. The FA of the whole WM skeleton was lower in TBI patients than in age‐matched controls (*t* = –6.55, *D* = 1.32, *p* < 0.001). A tract‐based analysis of FA^39^ showed reductions of FA following TBI in a large number of WM tracts (Figure S1). FA across the whole WM skeleton was significantly positively correlated with the WM JD volume change rate (Spearman's *R* = 0.37, *p* = 0.002, Figure S2). Furthermore, mean tract FA was a significant predictor of tract JD (*Z*‐scored, adjusted *R* ^2^ = 0.43, *p* = 0.023), demonstrating that those tracts more affected by diffuse axonal injury (lower FA) showed more longitudinal atrophy (more negative JD) (Figure S3).

In contrast to the finding that baseline FA predicted WM atrophy, there was no significant correlation (*p* > 0.05) between time since injury and atrophy. This remained the case when the group was split by the presence of focal lesions.

## 4 DISCUSSION

Here, we showed that patterns of progressive brain atrophy in the chronic phase after single moderate to severe TBI are highly distinctive, differing significantly from both mild to moderate late‐onset AD and healthy brain aging. Patients after TBI had rates of atrophy in WM and GM similar to those of HCs several decades older, although rates were greatest in patients with AD. The involvement of central WM structures, particularly the corpus callosum, internal capsules, and corticospinal tracts, was specific to TBI and not found in AD or aging. Conversely, progressive cortical atrophy was not widely present after trauma, with greater specificity to AD. Progressive atrophy patterns after moderate to severe TBI had not been previously directly compared with AD, particularly within the WM.

Axonal injury caused by TBI can trigger neurodegeneration. Progressive atrophy is observed following TBI, particularly in WM,^13^ and the spatial pattern and extent of post‐traumatic axonal injury predicts neurodegeneration over time.^19^ Here, the distinctive distribution of TBI‐specific atrophy was highly reminiscent of common patterns of axonal injury reported after head injury, attributable to shear forces affecting central WM structures, and was predicted by DTI measures of axonal injury.^40^ Given the substantial atrophy rates previously reported after moderate to severe TBI,^14^ this study assessed similarities with AD/aging and a group of moderate to severe rather than mild patients. This was motivated by a desire to maximize effect sizes (ie, of atrophy after injury) to maximize power to detect disease‐specific change when contrasting the groups. Indeed, the importance of injury severity is apparent in our cohort: Baseline WM integrity (FA) predicted atrophy, and atrophy rates were greater in injuries with lower conscious levels early after injury.

We found no clear relationship between atrophy rates and time after injury, suggesting atrophy is not solely a manifestation of slow Wallerian degeneration, which might be expected to decay in magnitude predictably over time. Neurodegeneration promoted by toxic spreading proteinopathies, such as of hyperphosphorylated tau, is a possible driver of post‐traumatic atrophy.^41^ It is notable that prion‐like tau seeding has been demonstrated in experimental injury models, potentially linking acute to chronic, progressive problems.^42^ Atrophy rates are most markedly elevated and change dynamically in the first 3 to 6 months after injury.^16^, ^43^ With baseline visits an average of 2 years after injury, our cohort was established in the early chronic phase. The magnitude of atrophy was stable over time, but changing spatial patterns cannot be excluded. Our TBI patients were typically in their fifth decade when assessed. Extended neuroimaging follow‐up over many decades within large TBI cohorts, such as the Initiative for Traumatic Brain Injury Research (InTBIR) studies, would allow this to be clarified.

There was a high degree of spatial overlap in patterns of brain volume reduction at baseline in AD and healthy aging. Aging is the foremost risk factor for a range of late‐onset neurodegenerative disorders including AD,^44^ and brain volume loss is a feature of both neurodegenerative dementias and aging.^45^, ^46^ Our finding of substantial spatial similarity in volume loss between aging and AD is in keeping with previous work, although we did not clearly demonstrate aging‐specific frontal atrophy.^47^ However, our aging group did not undergo biomarker evaluation to exclude the possibility of presymptomatic AD, which may have reduced our sensitivity.

We did not find spatial congruence between atrophy patterns over time in TBI and aging. Indeed, after removing regions of age‐related brain atrophy, there remained a clear TBI‐specific pattern of progressive atrophy involving the corpus callosum and corticospinal tracts. Machine‐learning approaches to volumetric MRI analysis in neurodegenerative disease have suggested that brains in the chronic phase after TBI appear older than would be expected^48^ and that this is also the case in patients with AD.^49^ Our study, however, suggests that apparent increases in “brain age” after TBI are likely to be driven by tissue loss in locations distinct from those characterizing healthy aging.

An improved understanding of the distinctive atrophy patterns after single moderate to severe TBI has potential to aid diagnosis where there is concern about possible AD alongside a history of significant neurotrauma and/or in advanced age. Neuroradiology clinical reporting of MRI data might usefully assess corticospinal/callosal atrophy for this purpose and computational comparison of individual patient data against large normative datasets may provide yet further specificity in the future.^50^ Machine learning models trained on our data would require testing in larger, diverse external datasets to ensure validity and generalizability.

This study has several potential limitations. Brain atrophy is a nonspecific measure of neurodegeneration, which limits pathological inferences, although spatial patterns of atrophy do carry some information about underlying neuropathology, for example, the strong correlation of tau but not amyloid pathology with atrophy in AD.^24^ For post‐traumatic atrophy, the lack of specificity limits the ability to distinguish between Wallerian degeneration triggered by initial axonal injury and progressive proteinopathy that accelerates or interacts with that seen in other neurodegenerative conditions. Although sex was included as a regressor in the analyses, we cannot exclude the possibility of bias arising from the greater proportion of men in the TBI group compared with age‐matched controls. However, hierarchical partitioning of variance has shown that sex explains relatively little variance in WM atrophy rates (independent *R* ^2^ = 0.3%) compared with the presence or absence of TBI (*R* ^2^ = 19.8).^14^ We tried to account for age differences between patients with TBI and those with late‐onset AD using multiple age‐matched control groups. However, this approach does not control for all the potentially confounding effects of age in the comparison of older AD and younger TBI patients. Comparing an aged TBI cohort following early‐ to mid‐life TBI with sporadic AD in older age and/or an early‐onset AD group with a young to middle‐aged TBI would help in future research to hold as many variables constant as possible across comparison groups. Imaging data were acquired at two sites on different scanners, with minor differences in acquisition sequences (Section 2), but, importantly, each participant was imaged longitudinally on the same system, acting as their own “control” and mitigating the effects of scanner differences. This is therefore unlikely to have introduced significant bias when comparing the groups.^35^

The presence of focal lesions may have influenced our findings, but we do not suspect a significant effect on these conclusions. For example, Cole et al. previously demonstrated no significant influence on WM atrophy rates of focal lesions using longitudinal volumetric MRI an average of 12 months after injury (nor was there any relationship between time since injury and WM atrophy rates).^14^ The overall presence or absence of focal lesions was included as a nuisance regressor in voxelwise analyses to mitigate this. TBI patients were established within the chronic phase at approximately 2 years following an event, making significant ongoing evolution of focal lesions or resolution of oedema unlikely. In the Hayes study of cortical atrophy in patients at high genetic risk for AD, it is notable that the mean time since injury was greater than within our cohort, although genetic stratification of the group may have been the main contributor to sensitivity in this work.^51^ A lack of genetic data, including APOE status, meant we were unable to assess for an interaction with atrophy. Socioeconomic status, ethnicity, and educational background were not systematically captured in the study; hence, we were unable to assess the influence of these factors on brain atrophy. Finally, we did not have a control group comprising patients with traumatic extracranial injuries in the absence of TBI. Given that non‐TBI trauma may affect the brain,^52^ prospective inclusion of this group in future work would help to demonstrate the specificity of our findings. In addition, diffusion imaging performed across AD and other control groups would confirm the specificity of the TBI‐specific atrophy involving callosal and corticospinal regions.

One measure of the severity of TBI that is particularly relevant to the neurodegeneration of WM tracts is the degree of axonal injury within the tract. Progressive atrophy is observed following TBI, particularly in WM,^13^ and the spatial pattern and extent of post‐traumatic axonal injury predicts neurodegeneration over time.^19^ Here we show again that the amount of atrophy in a WM tract following TBI correlates with the extent of axonal injury to that tract, measured by diffusion MRI.^40^ This suggests that tracts exposed to high shear forces at the time of injury go on to progressively atrophy for many years after TBI. We focused our analysis on moderate to severe TBI to reduce variability in the degree of post‐traumatic atrophy related to very minor injuries. We did not subdivide the severity of injury in the moderate to severe group on clinical grounds because this is methodologically challenging to do.^53^ One approach would be to choose one of the contributing factors to the classification, for example Glasgow Coma Scale, and use this as a proxy for severity, but individual clinical measures are noisy measures of injury severity, limiting the value of this type of analysis. More generally, the importance of injury severity is apparent in the strong relationship between axonal injury to WM tracts and subsequent atrophy rates. Diffusion imaging was not available for AD and all control groups. Hence, further research will be needed to investigate whether the relationship between diffusion measures of axonal injury and atrophy is specific to TBI; though this has been shown in midlife,^54^ it has not, to our knowledge, been tested in aging or AD.

In conclusion, we show that post‐traumatic neurodegeneration 1.9 to 4.0 years (median) after a single moderate to severe TBI, is distinct from AD and healthy aging, which showed similarities. Patterns of volume loss within the deep cerebral WM are reminiscent of typical patterns of diffuse axonal injury and appear specific to TBI. Indicating the magnitude of brain atrophy after TBI, we show that rates are similar after injury to healthy individuals 3 decades older. This work could be extended in future studies by characterizing the molecular basis of post‐traumatic atrophy using aligned positron emission tomography and blood biomarker assessment. A better understanding of the relationship between injury and progressive sequelae would help to focus strategies to prevent dementia after TBI and to target clinical trials of anti‐neurodegenerative treatments, which could make use of brain atrophy rates as an outcome measure.

## AUTHOR CONTRIBUTIONS

Neil S.N. Graham, James H. Cole, Jonathan M. Schott, and David J. Sharp conceptualized the research and contributed to the analysis plan. Neil S.N. Graham performed the review and neuroimaging analyses and wrote the original draft. Niall Bourke performed the diffusion analyses. All authors reviewed, edited, and approved the final manuscript.

## CONFLICT OF INTEREST

DJS declares membership of the UK RFU expert committee on concussion. JMS is Chief Medical Office for ARUK and serves on the research committee for the Football Association. NSNG and JC declare no potential conflicts of interest. Author disclosures are available in the supporting information.

## CONSENT STATEMENT

All human subjects provided informed consent.

## Supporting information

Supporting Information

Supporting Information
