# Supplementary material for: Distinct patterns of neurodegeneration after TBI and in Alzheimer's disease
Source: Alzheimers Dement. 2023 Jan 25;19(7):3065–77. doi: 10.1002/alz.12934 (PMC10955776; doi:10.1002/alz.12934)
Supplement: Supplementary file 1 — Supporting Information [file ALZ-19-3065-s002.pdf]

Distinct patterns of neurodegeneration after TBI and in Alzheimer's disease

Neil SN Graham, James H Cole, Niall J Bourke, Jonathan M Schott, David J Sharp

SUPPLEMENTARY

METHODS

IMAGE ACQUISITION

UCL, patients were assessed on a GE medical systems 1.5T system. An inversion recovery prepared fast spoiled gradient recalled (IR-FSPGR) sequence was used to acquire 3D volumetric T1-weighted images in AD patients and age-matched controls longitudinally with a field of view of 24 cm, with a time to repetition of 15ms, an echo time of 5.4ms, TI of 650ms, flip angle of 15°, in a 256x256 matrix, comprising 124 contiguous 1.5 mm coronal slices, resulting in a voxel size of 0.9735mm x 0.9735mm x 1.5mm.

At ICL, patients were assessed twice on the same Siemens Verio 3T system (Siemens Healthineers AG, Erlangen, Germany), alongside age-matched controls and a group of older healthy controls to assess the effect of ageing on brain structure. The MPRAGE sequence used the following parameters: slice thickness of 1mm, with 160 slices and a matrix of 256x240, with a repetition time of 2300ms, echo time of 2.98ms, flip angle 9°, field of view of 25.6cm x 24cm, with a voxel size of 1.0 x 1.0 x 1.0 mm. Diffusion MRI was available at baseline for the majority of the ICL TBI patients and age-matched healthy controls. Diffusion weighted images were acquired on the 3T Siemens Verio system in 64 directions with an isotropic voxel size of 2mm3, b = 1000 s/mm3, and with four images where b = 0 s/mm2.

DIFFUSION TENSOR IMAGING ANALYSES

DTI analyses were performed according to standard approaches.[1] In brief, visual quality control checks were performed. Scans were processed using the tract based spatial statistics (TBSS) pipeline (FSL, Oxford, version 5.0.8),[2] with HD-BET used for improved brain extraction (Heidelberg),[3] and DTI-TK for generation of and tensor-based non-linear registration of individual data to a study template.[4] A white matter skeleton was generated by thresholding the group mean fractional anisotropy map at 0.2, and subject FA data were projected onto this skeleton.

RESULTS

Supplementary Figure 1. Widespread baseline reductions in FA across white matter tracts after TBI, versus healthy controls

Mean fractional anisotropy (FA) across ten tract regions of interest and a whole white matter skeleton average FA, in patients (shown in purple) and age-matched healthy volunteers (yellow) after moderate-severe TBI. Fascic.: fasciculus; WM: white matter.

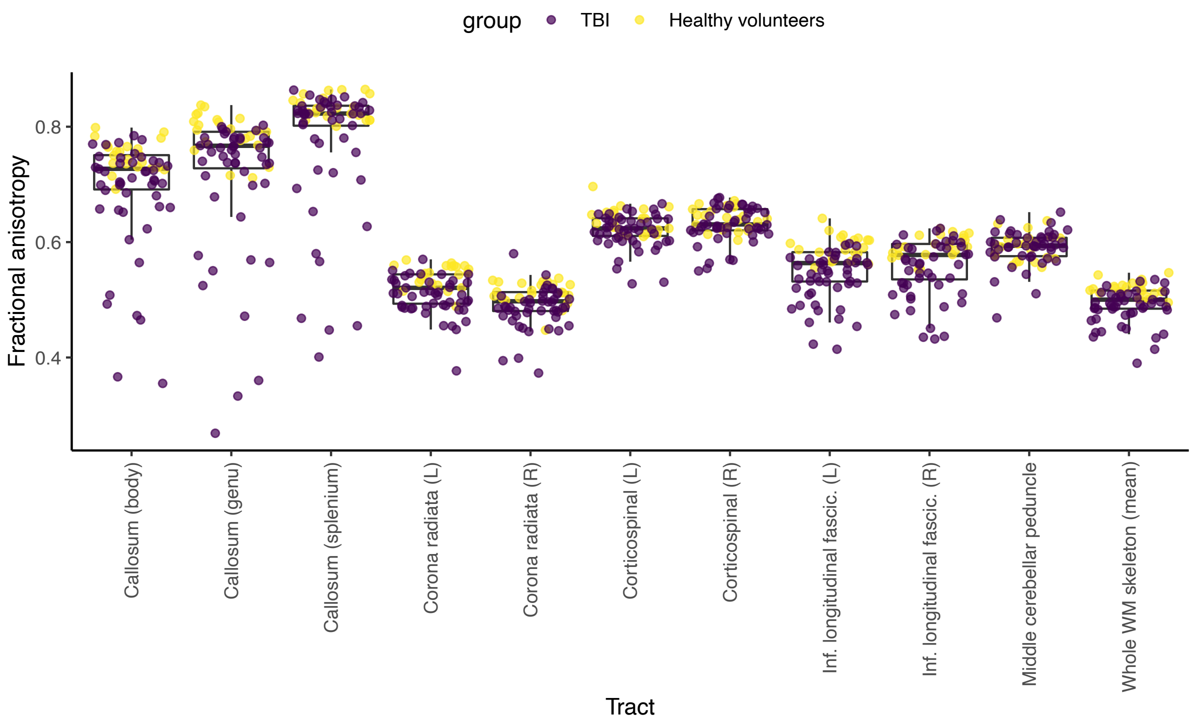

Supplementary Figure 2. Relationship between white matter integrity at baseline and atrophy over time.

Mean white matter skeleton fractional anisotropy (FA) is positively correlated with the Jacobian Determinant (JD) annualised brain volume change rate (%) in white matter.

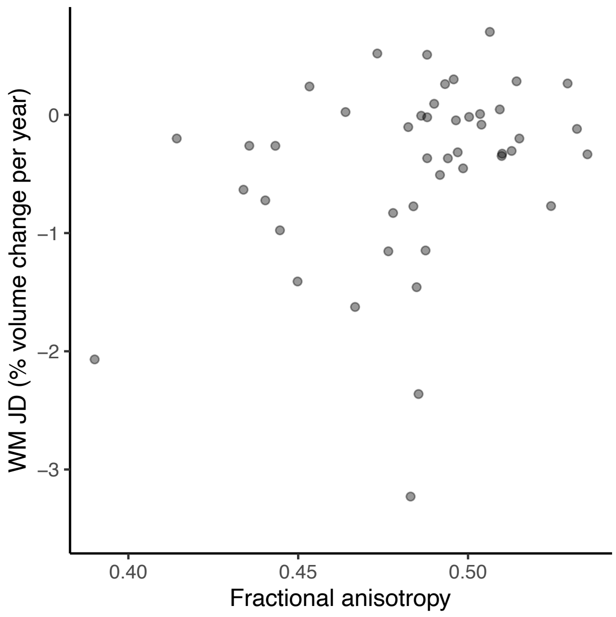

Supplementary Figure 3. Relationship between white matter integrity and progressive atrophy across different tracts

Fractional anisotropy (FA, z-scored against aged matched healthy controls) predicts Jacobian determinant white matter volume change rate (JD, z-scored) across ten tract regions of interest. Inf.=inferior; Fasc.=fasciculus; (R)=right; (L)=left.

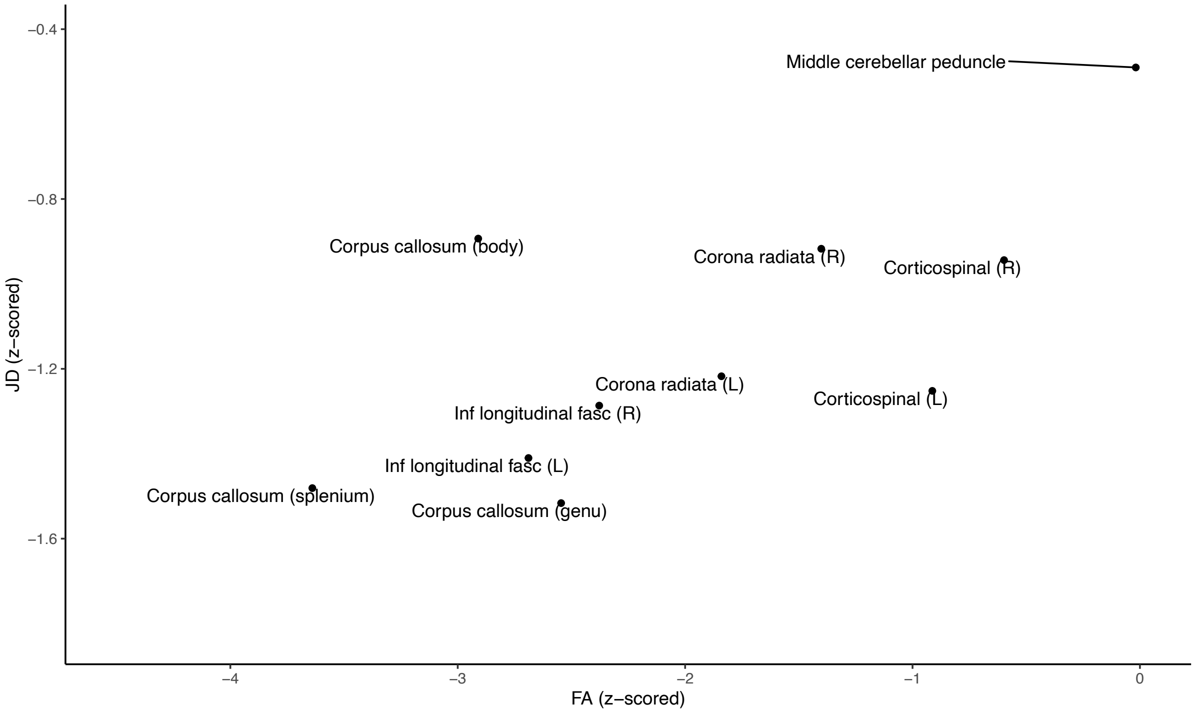

SUPPLEMENTARY REFERENCES

1. Graham NSN, Jolly A, Zimmerman K, et al. Diffuse axonal injury predicts neurodegeneration after moderate–severe traumatic brain injury. *Brain*. 2020;doi:10.1093/brain/awaa316

2. Smith SM, Jenkinson M, Johansen-Berg H, et al. Tract-based spatial statistics: voxelwise analysis of multi-subject diffusion data. *Neuroimage*. Jul 15 2006;31(4):1487-505. doi:10.1016/j.neuroimage.2006.02.024

3. Isensee F, Schell M, Pflueger I, et al. Automated brain extraction of multisequence MRI using artificial neural networks. *Human Brain Mapping*. 2019;40(17):4952-4964. doi:https://doi.org/10.1002/hbm.24750

4. Zhang H, Avants BB, Yushkevich PA, et al. High-dimensional spatial normalization of diffusion tensor images improves the detection of white matter differences: an example study using amyotrophic lateral sclerosis. *IEEE Trans Med Imaging*. Nov 2007;26(11):1585-97. doi:10.1109/TMI.2007.906784
